# Ibrutinib-induced acute kidney injury via interstitial nephritis

**DOI:** 10.1080/0886022X.2021.1874985

**Published:** 2021-02-10

**Authors:** Csilla Markóth, Ibolya File, Róbert Szász, László Bidiga, József Balla, János Mátyus

**Affiliations:** a Division of Nephrology, Department of Internal Medicine, Faculty of Medicine, University of Debrecen, Debrecen, Hungary; b Division of Hematology, Department of Internal Medicine, Faculty of Medicine, University of Debrecen, Debrecen, Hungary; c Department of Pathology, Faculty of Medicine, University of Debrecen, Debrecen, Hungary

**Keywords:** Acute kidney injury, acute interstitial nephritis, chronic lymphocytic leukemia, ibrutinib, cytokines, glucocorticoids

## Abstract

The introduction of Bruton's tyrosine kinase inhibitor ibrutinib has made a significant progress in the treatment of chronic lymphocytic leukemia and other B-cell malignancies. Due to the reduction of cytokine release, it is effective in chronic graft-versus-host disease, and its use has also been suggested in autoimmune diseases and in prevention of COVID-19-associated lung damage. Despite this effect on the immune response, we report a severe hypersensitivity reaction in a 76-year-old male patient diagnosed with prolymphocytic leukemia. Four weeks after the ibrutinib start, non-oliguric acute kidney injury with proteinuria and microscopic hematuria developed and that was accompanied by lower limb purpuras and paresthesia. Renal biopsy revealed acute interstitial nephritis. Employing 1 mg/kg methylprednisolone administration, serum creatinine decreased from 365 μmol/L to 125 μmol/L at 11 days and the proteinuria-hematuria as well as the purpura, paresthesia resolved. Three months later at stabile eGFR of 56 ml/min/1.73 m^2^ methylprednisolone was withdrawn and a rituximab-venetoclax treatment was initiated without side effects. We conclude that despite the beneficial effect on cytokines response in Th1 direction, ibrutinib can cause acute interstitial nephritis. Early detection, discontinuation of ibrutinib, glucocorticoid administration may help to better preserve renal function, thereby lowering the risk of potential subsequent kidney injury.

## Introduction

The Bruton's tyrosine kinase (BTK) is an essential B-cell antigen receptor signaling molecule for B-cell development and activation pathway, which is involved in the pathogenesis of several B-cell malignancies. Ibrutinib, the first representative of BTK inhibitors, significantly improved the prognosis in high genetic risk chronic lymphocytic leukemia (CLL) resistant to traditional chemoimmunotherapy [1], in mantle cell lymphoma and in Waldenström’s macroglobulinemia as well.

BTK is required for the normal function of immune cells other than B cells, it controls cytokine production, phagocytosis, and the formation of inflammatory mediators. Ibrutinib treatment improves immune dysfunctions associated with CLL, the nonmalignant B-cell immune repertoire remains stable, T-cell and myeloid cell defects are partially restored [2–4]. Irreversible inhibition of the BTK-homologous interleukin-2-inducible T-cell kinase also contributes to this, stimulation of which is involved in selective Th2 cell activation, directing the immune response to healthy tissues. Therefore, ibrutinib also significantly improved the steroid-resistant/dependent chronic graft-versus-host disease [5]. It may be effective in preventing COVID-19-induced lung injury and may even improve the hypoxic, coronavirus-infected individuals’ lung function [6]. Reduction of cytokine release syndrome has also been observed in CLL patients receiving ibrutinib prior to obinutuzumab infusion [7]. Due to additive effects beyond B-cell depletion, BTK inhibition appears promising also in autoimmune diseases [8,9].

The more widespread use of ibrutinib is due to being a well-tolerated, orally applicable drug. Its most common side effects (diarrhea, skin hemorrhages, hypertension, upper respiratory tract infections) are usually mild; rarely, more severe bleeding complications or atrial fibrillation may occur as grade 3/4 side effects. It very rarely causes tumor lysis syndrome [10] or other kidney injuries in itself, not mentioned in the recent summary of adverse reactions [11], nor has been observed in a 6-year follow-up [12]. The acute kidney injury (AKI) cases reported in a previous trial were due to preceding renal diseases and concomitant pre/postrenal reasons [13]. In two renal biopsy cases degenerative tubular damage was assumed as a consequence of long-term ibrutinib administration [14].

In our patient, 4 weeks after ibrutinib start, acute interstitial nephritis (AIN) was recognized during investigations for lower limb purpura and neuropathic pain. The occurrence of a severe hypersensitivity reaction is unexpected given the ‘beneficial’ effect of ibrutinib on cytokines, no similar case was reported.

## Case report

**Figure 1.**
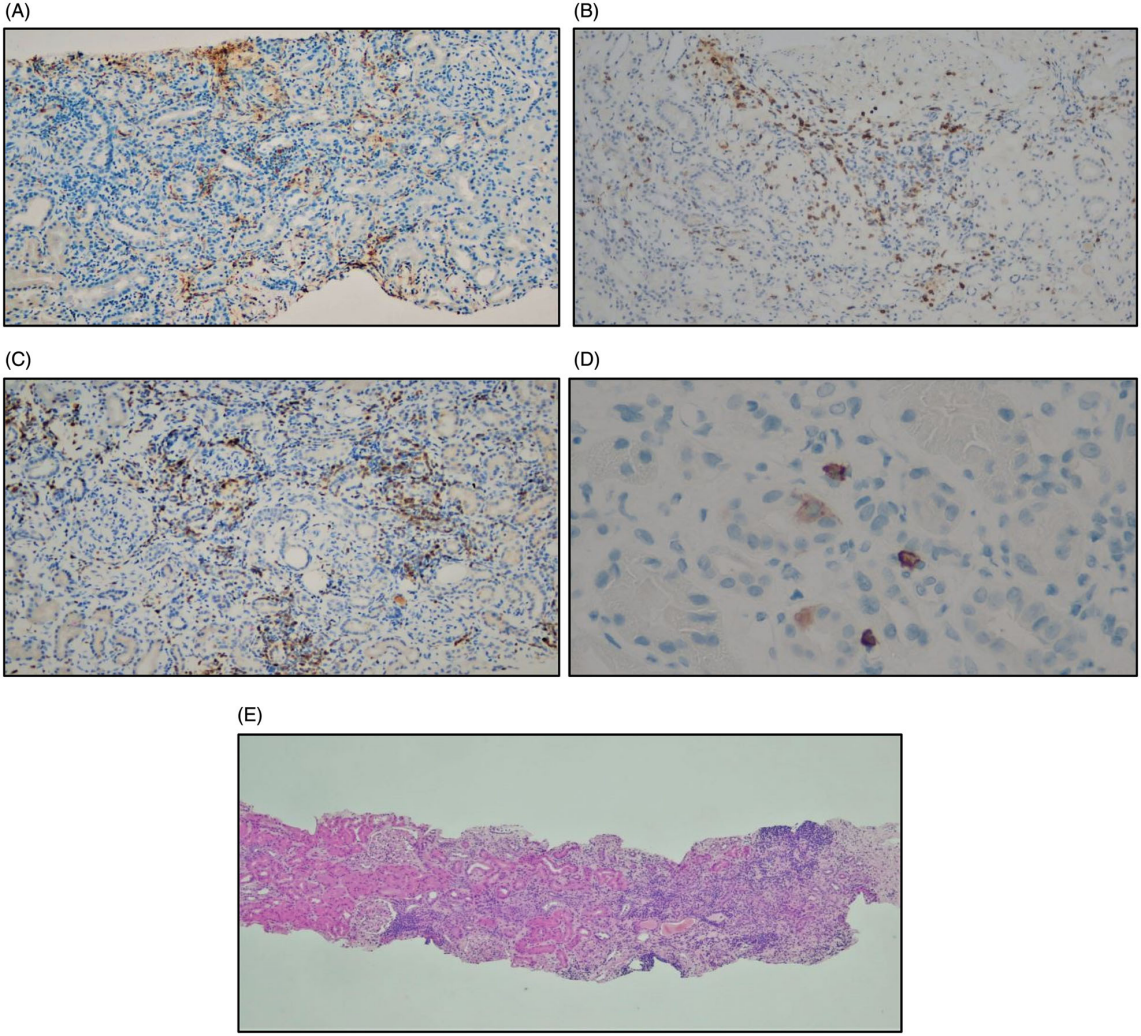
Kidney biopsy specimen, monoclonal interstitial infiltration cannot be confirmed. (A) CD68-positive macrophages (100×), (B) CD20-positive B cells (100×), (C) CD3-positive T cells (100×), (D) CD117-positive mastocytes in the interstitium (3 cells on the right side of the FOV), (E) HE (40×) focal, mixed inflammatory cell infiltration in the interstitium

A 76-year-old man with a history of depression, hypertension, previous regular smoking (half pack/day for 20 years) and alcohol consumption (5 unit/day for 30 years) presented with AKI. In January 2018, close hematological monitoring was started due to high white blood cell count (42 G/L, predominantly lymphocytes 32 G/L). By peripheral flow cytometry, 77% of the B cells proved to be abnormal, the immunophenotype raised the possibility of prolymphocytic leukemic transformation. In January 2020 white blood cell count increased to 116 G/L, CT scan showed significant splenomegaly, paraaortic lymph node enlargement, with serum creatinine (sCr) of 59 μmol/L at that time. Because of progression of the underlying disease, with prognostic markers including monosomy 13, TP53 (17p13.1) deletion, and borderline mutation status of immunoglobulin heavy chain variable region, ibrutinib was initiated in March 2020 at a daily dose of 420 mg. Previous medications (tianeptine, enalapril, spironolactone, diosmin) were continued except aspirin. Four weeks later, he was referred to the Emergency Department because of lower limb purpuras, burning pain in both feet and soles. A significant sCr increase (296 μmol/L) was discovered. On the following day, despite preserved urinary output, sCr increased to 365 μmol/L, with significant proteinuria (urine protein to creatinin ratio: 70 mg/mmol), and microscopic hematuria. No other abnormalities than moderate serum uric acid (537 μmol/L), and phosphate (1.8 mmol/L) elevations, a slightly decreasing white blood cell count (98 G/L), and anemia (hemoglobin 113 g/L) were detected. Serum potassium, tCO2, calcium, platelet and eosinophil cell counts, liver and muscle enzymes, immunoglobulin and complement levels were normal. Urgent anti-neutrophil cytoplasmic antibody (ANCA) and anti-glomerular basement membrane antibody test results were also negative. Ultrasonography showed normal-sized kidneys, without urine outflow obstruction. Because of rapidly deteriorating renal function, parenteral methylprednisolone was initiated at a dose of 1 mg/kg body weight, and ibrutinib was omitted. Renal biopsy confirmed acute interstitial nephritis, it showed no crescent formation, interstitial B-cell infiltration or other significant abnormalities on day 4 (Figure 1). Immunofluorescence revealed no immunodeposits in the glomeruli, the blood vessels, interstitium, tubules. Focal intense, interstitial infiltration of mixed inflammatory cells with some eosinophil cells and mastocytes were seen. Methylprednisolone treatment was continued, with which the patient’s skin symptoms, lower limb numbness were completely resolved, on day 11 of treatment, sCr was reduced to 125 μmol/L, when no proteinuria/hematuria was detectable. Apixaban was started due to atrial fibrillation occurring at this time and high stroke risk score. Methylprednisolone therapy was gradually tapered off over 3 months, and eGFR stabilized at 56 mL/min/1.73 m^2^. Due to further increase in white blood cell count (160 G/L), rituximab—venetoclax treatment was initiated in August 2020. Tumor lysis syndrome did not occur with rasburicase and allopurinol prophylaxis.

## Discussion

Significant progress has been made in the treatment of CLL over the past decade. In our patient, genetic tests clearly indicated a poor prognosis, thus according to the recommendations [1], ibrutinib was initiated at disease progression, after which, 4 weeks later, non-oliguric AKI occurred. Both pre/postrenal etiologies and tumor lysis syndrome were ruled out on the basis of the clinical picture, renal ultrasonography and laboratory tests. Proteinuria and microscopic hematuria raised the possibility of both glomerular and tubulointerstitial damage. Because sCr, urine tests were not performed before starting treatment, it could not be ruled out that renal involvement may be a rare consequence of CLL. Leukemic cell infiltration is usually associated with renal enlargement, while paraneoplastic glomerular diseases are often associated with nephrotic syndrome, which were all absent in our case. However, they can only be ruled out by kidney biopsy, which also enables us to distinguish among the various histological forms of involvement. In the Mayo Clinic’s 10 years of practice, renal biopsy was required in 1.2% of CLL patients due to kidney failure or nephrotic syndrome [15].

The coinciding skin symptoms and neuropathy a month after the initiation of ibrutinib made us think of its provocative role in the development of AKI. The discontinuation of a potentially life-saving treatment warranted a kidney biopsy. Ibrutinib inhibits of collage-induced platelet aggregation by inhibiting platelet BTK and it is generally recommended to hold it 3–7 days before and after and invasive interventions [11]. This resulted in waiting until day 4 to perform a renal biopsy to decrease the risk of bleeding from platelet dysfunction.

Thrombocytopathy may have a role in the formation of small, non-palpable petechias usually developing after 2 months of ibrutinib treatment. In our patient, the prompt presence of skin symptoms within one month of therapy was more suggestive of vasculitis. Such early onset of palpable purpuriform lesions may suggest more severe disease and may require discontinuation of treatment, glucocorticoid therapy, and could recur upon repeated administration of the drug [16]. Peripheral neuropathies due to various pathomechanisms are not uncommon in hematologic malignancies [17]. Ibrutinib may ameliorate anti-myelin-associated glycoprotein mediated form but can also provoke it [11,17] by an unknown mechanism. In our case, its simultaneous appearance with skin and kidney symptoms raised the possibility of vasculitis.

The related clinical picture abovemade drug-induced interstitial nephritis higher on the differential over IgA or ANCA induced vasculitis. The rapid loss in GFR prompted us to start glucocorticoid immediately prior to any histological verification along with holding ibrutinib. Renal biopsy confirmed our suspicions. One prior case showed biopsy-proven ibrutinib induced AIN to date; however, in that case, chronic kidney disease and diarrhea were also implicated in the AKI [18].

In the background of drug-induced AIN, a hypersensitive reaction is suggested, primarily characterized by the Th2 cytokine response. Its occurrence is unusual because ibrutinib can prevent cytokine release in CLL patients [6,7], decreases the elevated cytokine, chemokine levels and polarizes T cells in Th1 direction [3]. Macrophage response shifts from M1 toward M2 based on mouse models and human studies, too [4,6]. In our case, we did not examine cytokine levels. Recently serum and urine levels of several cytokines were found to be higher in AIN patients compared to healthy ones, but cytokine levels characteristic of Th2 response did not differ [19]. Because AIN is mostly restricted to the kidneys, testing urine cytokine levels is more relevant. Th2-specific TNF-alpha urinary level was higher in AIN compared to other AKI, but it can be mast cell origin, as indicated by higher IL-9 levels [20]. Mast cells were present in our case biopsy specimen also.

With glucocorticoid treatment, proteinuria, hematuria resolved within 2 weeks, and renal function improved significantly. Glucocorticoid treatment efficacy is contentious in AIN [19], and many consider it only if no improvement in renal function is observed within 3–5 days despite omitting the responsible drug. This wait would certainly have resulted in lower GFR later. In case of malignant diseases, we consider it important to better preserve kidney function also for the prevention of AKI that may be superimposed due to possible subsequent damages.

With this case report, we would like to draw the attention to careful use of ibrutinib, which certainly will be applied more widely. Publication of our case may also contribute to explore the pathomechanism of AIN.

## Abbreviations

AIN: acute interstitial nephritis
AKI: acute kidney injury
BTK: Bruton's tyrosine kinase
CLL: chronic lymphocytic leukemia
sCr: serum creatinine
GFR: glomerular filtration ratio
ANCA: anti-neutrophil cytoplasmic antibody
